# A new Peruvian species of *Scybalocanthon* Martínez, 1948 (Coleoptera, Scarabaeidae, Scarabaeinae, Deltochilini) and some remarkable intrapopulational variation in the endophallus of *S.pinopterus* (Kirsch, 1873)

**DOI:** 10.3897/zookeys.884.39322

**Published:** 2019-10-30

**Authors:** Fernando A. B. Silva, François Génier

**Affiliations:** 1 Universidade Federal do Pará, Instituto de Ciências Biológicas, setor de Zoologia. Rua Augusto Corrêa, 01. Guamá. Belém PA 66075-110, Brazil Universidade Federal do Pará Belém Brazil; 2 Beaty Centre for Species Discovery, Canadian Museum of Nature, P.O. Box 3443, Station D, Ottawa, Ontario K1P 6P4, Canada Canadian Museum of Nature Ottawa Canada

**Keywords:** Dung beetles, neotropical fauna, taxonomy

## Abstract

*Scybalocanthonashei***sp. nov.** from Madre de Dios, Peru, is described based on differences in external and male genital morphology. Its diagnostic characters and an updated identification key to the species of the genus are provided as well as new distributional data for the following species: *S.acrianus* Silva & Valois, 2019, *S.aereus* (Schmidt, 1922), *S.kaestneri* (Balthasar, 1939) and *S.pinopterus* (Kirsch, 1873).

## Introduction

The genus *Scybalocanthon* Martínez, 1948 comprises species distributed from Costa Rica to northern Argentina (Pereira and Martínez 1956; Silva and Valois 2019). The genus was recently revised by Silva and Valois (2019), who recognized 23 valid species: *S.acrianus* Silva & Valois, 2019; *S.adisi* Silva & Valois, 2019; *S.aereus* (Schmidt, 1922); *S.arnaudi* Silva & Valois, 2019; *S.batesi* Vaz-de-Mello & Silva, 2017; *S.chamorroi* Silva & Valois, 2019; *S.cyanocephalus* (Harold, 1868); *S.darlingtoni* (Paulian, 1939); *S.federicoescobari* Silva & Valois, 2019; *S.haroldi* Silva & Valois, 2019; *S.kaestneri* (Balthasar, 1939); *S.kelleri* Pereira & Martínez, 1956; *S.korasakiae* Silva, 2011; *S.maculatus* (Schmidt, 1920); *S.martinezi* Silva & Valois, 2019; *S.moniliatus* (Bates, 1887); *S.nigriceps* (Harold, 1868); *S.papaxibe* Silva & Valois, 2019; *S.pinopterus* (Kirsch, 1873); *S.pygidialis* (Schmidt, 1922); *S.sexspilotus* (Guérin-Méneville, 1855); *S.trimaculatus* (Schmidt, 1922); and *S.uniplagiatus* (Schmidt, 1922) (see Silva and Valois 2019 for taxonomic background).

According to the current definition of the genus, *Scybalocanthon* is characterized by the first meso- and metatarsomeres with external margin with one-half the length of the second tarsomeres, and with apex obliquely truncated; lateral borders of tarsomeres parallel, forming a continuous border for all tarsomeres; overall shape of tarsomeres 2‒4 square to rectangular; and dorsal surface of mesotibiae with dense, randomly distributed setae (Silva and Valois 2019).

Examination of the collection at the Canadian Museum of Nature (CMNC), Ottawa, has revealed a new species. In this paper, we describe this newly discovered species and provide an updated key to the species of *Scybalocanthon*. Besides, we also present new distributional data for *S.acrianus*, *S.aereus*, *S.kaestneri* and *S.pinopterus*. The results also show remarkable intrapopulational variations in the endophallus of *S.pinopterus*.

## Materials and methods

The material studied was deposited in the following collections: **CEMT/UFMT** (Seção de Entomologia da Coleção Zoológica, Cuiabá, curator Fernando Vaz-de-Mello); **CMNC** (Canadian Museum of Nature, Ottawa, Canada, curator François Génier); **MZUFPA** (Coleção de Scarabaeinae do Museu de Zoologia, Instituto de Ciências Biológicas, Universidade Federal do Pará, Belém, Brazil, curator Fernando A. B. Silva).

Examination of the aedeagus and endophallic sclerites allowed clarifying the differences between species. In describing these structures, we followed Tarasov and Solodovnikov (2011) and Tarasov and Génier (2015). The endophallus was removed from the aedeagus through the basal foramen of the phallobase, and its sclerites were illustrated in ventral view, except for the superior right peripheral sclerite (**SRP**), which was illustrated from the right side of the aedeagus. The following sclerites were found to be taxonomically useful: Superior Right Peripheral Sclerite (**SRP**) and Fronto-Lateral Peripheral Sclerite (**FLP**).

Images of specimens and key characters were taken with Leica stereomicroscope M205A, using image stacking software (Leica Application Suite, version 3.7.0), and they were edited using Adobe Photoshop CS4.

## Results

### Taxonomy

#### Scybalocanthon ashei sp. nov.

Taxon classificationAnimaliaColeopteraScarabaeidae

656E1DFA-1C07-5C7B-A80D-56082C7957BE

http://zoobank.org/9E58A29A-B2F0-4893-8AF4-5EAA15821193

Figs 1A–G , 5C

##### Material studied.

***Holotype*.** PERU: MADRE DE DIOS, Reserva Cuzco Amazonica, 15 km NE Puerto Maldonado, 69°03'W, 12°33'S, 200m, 4.VII.1989, Ashe and Leschen legs (1♂ CMNC). ***Paratypes*** [5♂ and 7♀]. PERU: MADRE DE DIOS, Reserva Cuzco Amazonica, 15 km NE Puerto Maldonado, 69°03'W, 12°33'S, 200m, 7.VII.1989, Ashe and Leschen legs (1♀ CMNC); Reserva Cuzco Amazonica, 15 km NE Puerto Maldonado, 69°03'W, 12°33'S, 200m, 13.VII.1989, Ashe and Leschen legs (1♀ CMNC); Reserva Cuzco Amazonica, 15 km NE Puerto Maldonado, 69°03'W, 12°33'S, 200m, 17.VI.1989, Ashe and Leschen legs (1♀ CMNC); Reserva Cuzco Amazonica, 15 km NE Puerto Maldonado, 69°03'W, 12°33'S, 200m, 24.VI.1989, Ashe and Leschen legs (1♀ CMNC); Reserva Cuzco Amazonica, 15 km NE Puerto Maldonado, 69°03'W, 12°33'S, 200m, 26.VI.1989, Ashe and Leschen legs (1♂ CMNC, 1♂ MZUFPA, 1♂ CEMT); Reserva Cuzco Amazonica, 15 km NE Puerto Maldonado, 69°03'W, 12°33'S, 200m, 20.VI.1989, Ashe and Leschen legs (1♂ CMNC); Reserva Cuzco Amazonica, 15 km NE Puerto Maldonado, 69°03'W, 12°33'S, 200m, 30.VI.1989, Ashe and Leschen legs (1♀ CMNC); Parque Nacional del Manú, 15–30.VIII.1986, A. Forsyth leg. (1♂ and 1♀ CMNC, 1♀ MZUFPA).

##### Diagnosis.

**Figure 1.**
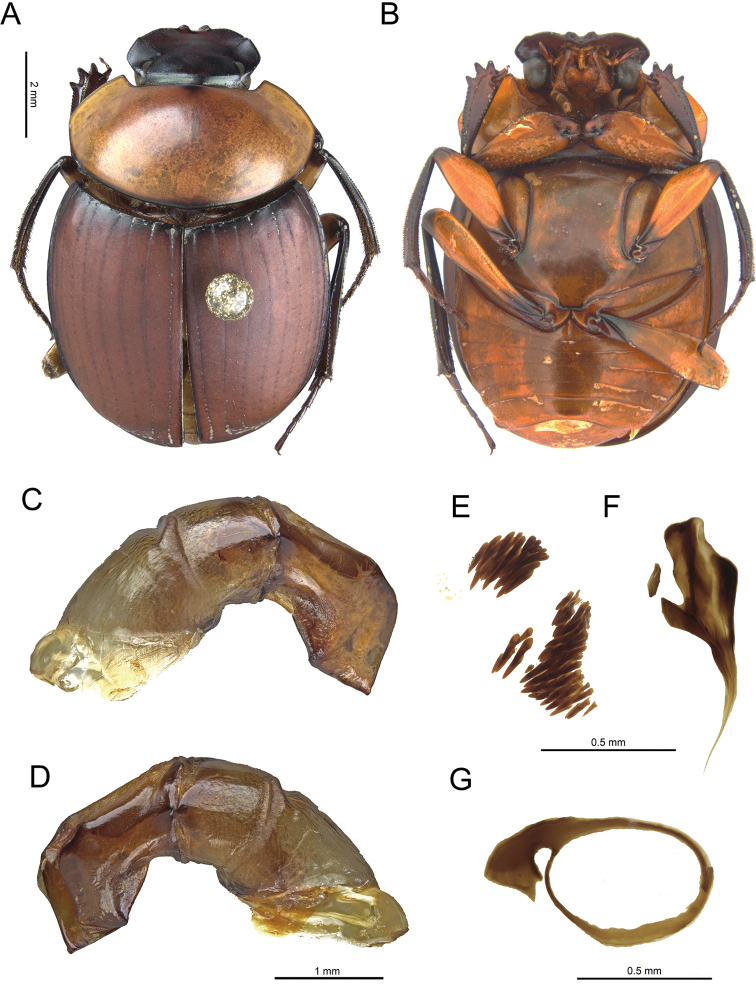
*Scybalocanthonashei* sp. nov. **A** Holotype (CMNC), dorsal view **B** holotype (CMNC), ventral view **C** aedeagus (right side) **D** aedeagus (left side) **E** set of bristles **F** fronto-lateral peripheral (FLP) sclerite (left), and Complex of axial and subaxial (A+SA) sclerites (right) **G** Superior right peripheral (SRP) sclerite.

**Figure 2.**
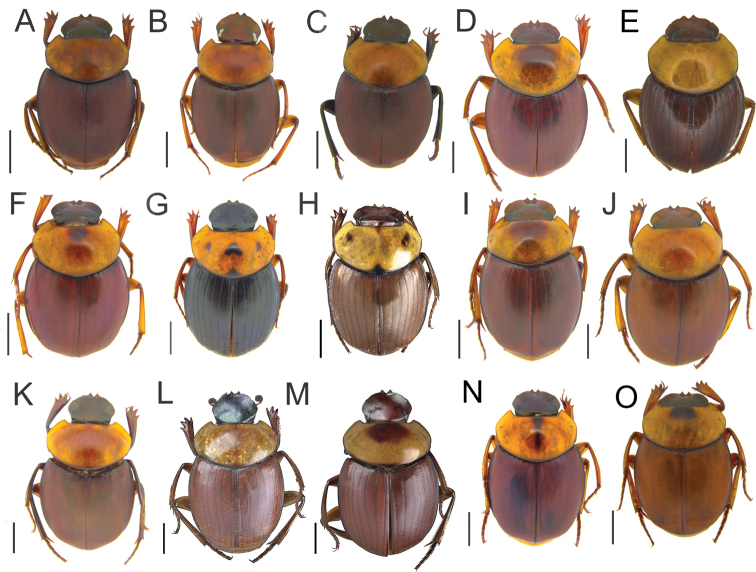
Species of *Scybalocanthon*, dorsal views. **A***S.acrianus***B***S.adisi***C***S.aereus***D***S.arnaudi***E***S.chamorroi***F***S.federicoescobari***G***S.kaestneri* (specimen from Pastaza, Ecuador) **H***S.kaestneri* (specimen from Napo, Ecuador) **I***S.martinezi***J***S.papaxibe***K***S.pinopterus* (specimen from Madre de Dios, Peru) **L***S.pinopterus* (specimen from Tingo Maria, Huanuco, Peru) **M***S.pinopterus* (specimen from Orellana, Ecuador) **N***S.pygidialis***O***S.uniplagiatus*, Scale bars: 2 mm.

**Figure 3.**
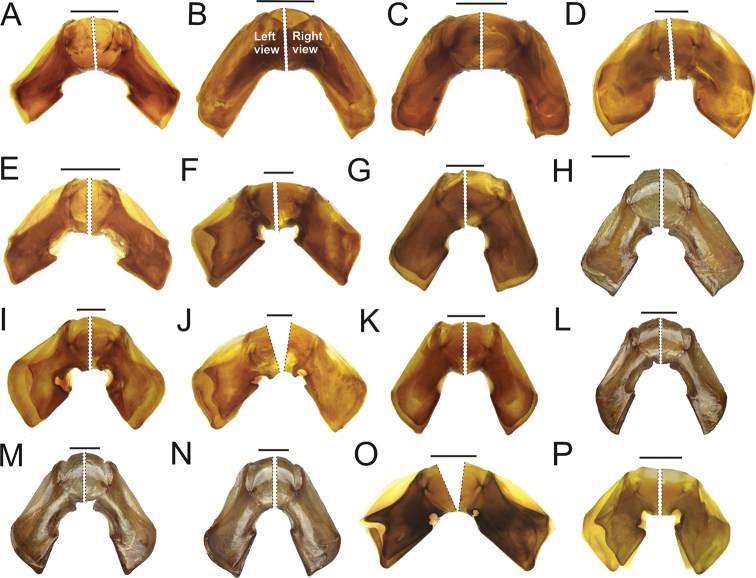
Aedeagus, detail of parameres in left and right view. **A***S.acrianus***B***S.adisi***C***S.aereus***D***S.arnaudi***E***S.chamorroi***F***S.federicoescobari***G***S.kaestneri* (specimen from Pastaza, Ecuador) **H***S.kaestneri* (specimen from Napo, Ecuador) **I***S.martinezi***J***S.papaxibe***K***S.pinopterus* (specimen from Madre de Dios, Peru) **L***S.pinopterus* (specimen from Tingo Maria, Huanuco, Peru) **M***S.pinopterus* (specimen from Tingo Maria, Huanuco, Peru) **N***S.pinopterus* (specimen from Orellana, Ecuador) **O***S.pygidialis***P***S.uniplagiatus*, Scale bars: 0.5 mm.

Specimens of *Scybalocanthonashei* sp. nov. (Fig. 1A) are similar to those of *S.arnaudi* (Fig. 2D), *S.federicoescobari* (Fig. 2F), *S.martinezi* (Fig. 2I), *S.papaxibe* (Fig. 2J), *S.pinopterus* (Fig. 2K–M), and *S.uniplagiatus* (Fig. 2O) in having the pronotum uniformly colored, or with one elliptical spot on the central portion; femora almost completely yellow or brown, with black spots only on the tips (Fig. 1B); eighth elytral stria with thin carina anteriorly (see Silva and Valois 2019, fig. 3D); endophallus with bristles right beside the FLP sclerite (Fig. 1E); and additional sclerite (AS) absent. They can be distinguished from those of *S.arnaudi* and *S.pinopterus*, however, by the strongly asymmetrical parameres; left paramere with acute projection on dorsal margin and bilobate excavation on ventral margin (Fig. 1C, D) (other species with slightly asymmetrical parameres, lacking acute projection and bilobate excavation on dorsal and ventral margins (Fig. 3D, K–N); from those of *S.federicoescobari*, *S.martinezi*, *S.papaxibe*, and *S.uniplagiatus* by the bilobate excavation of the ventral margin of the left paramere wider, extending along two-third of the paramere margin (Fig. 1D) (in *S.federicoescobari* (Fig. 3F) and *S.papaxibe* (Fig. 3J) the excavation extending along one-fourth of the paramere margin; in *S.uniplagiatus* (Fig. 3P) the excavation extending along one-third of the paramere margin; in *S.martinezi* (Fig. 3I) the excavation is deeper, extending about one-half of the paramere margin).

##### Description.

***Body***: Oval-elongated (Fig. 1A). General surface opaque, completely microgranulated. Pronotum with silky sheen. ***Color***: Most of pronotum, hypomera (except internal and posterior margins), metaventrite, metepisterna, abdominal ventrites, pygidium, and middle of femora yellow or light brown. Head, internal and posterior margins of hypomera, prosternum, mesoventrite, mesepisterna, elytra, trochanters, proximal and distal portions of femora, tibiae and tarsomeres dark brown. ***Length***: 8.8–11.5 mm. ***Head***: Dorsal surface with fine microgranulation. Clypeal margin with two small, triangular central teeth. Eye comma-shaped in dorsal view. ***Thorax***: Pronotum twice as wide as long, anterior angles acute, directed forward. Pronotum usually with one brown rounded spot at the anterocentral portion; in some specimens, spot absent. Anterior angles of pronotum approximately 80°. Lateral margin strongly curved outward. ***Elytra***: Striae thin and shiny, punctures conspicuous. Eighth stria with a thin carina anteriorly. ***Legs***: Protibiae with three lateral teeth. Anterior and posterior edge of meso- and metafemora not margined. Mesotibiae smoothly arched toward body. Metatibiae almost straight. First meso- and metatarsomeres short, external margin half the length of second tarsomere, and obliquely truncated apically. Lateral margins of tarsomeres parallel, forming even margin along length of tarsus. Overall shape of meso- and metatarsomeres 2–4 varying from quadrate to rectangular. Dorsal (internal) surface of mesotibiae with dense setae, randomly distributed. ***Secondary sexual characters***: Females can be distinguished from males by the sixth abdominal ventrite longer than in males, and the anterocentral portion of sixth abdominal ventrite more swollen than the posterocentral portion in lateral view (males have, in general, the posterocentral portion more swollen). ***Genital capsule***: Parameres strongly asymmetrical (Fig. 1C, D). Dorsal margin of right paramere curved inward, apex obliquely truncated. Ventral margin of right paramere with a rounded excavation at the basal portion (Fig. 1C). Dorsal margin of left paramere curved inward from basal to medial portions, medial portion with a short and pointed projection, apex obliquely truncated (Fig. 1D). Ventral margin of left paramere with a bilobate excavation extending along two-third of the paramere margin; apical third obliquely truncated (Fig. 1D). ***Endophallus***: SRP circular, with rounded handle-shaped extension (Fig. 1G). FLP short (Fig. 1F, left), comma-shaped, with three sets of bristles (Fig. 1E) right beside it. A+SA with two superposed and elongate sclerites (Fig. 1F, right).

##### Etymology.

Named in honor of the late James S. Ashe, collector of most of the known specimens.

##### Habitat.

Amazon rainforest. Known from Peru (Fig. 5C). **Endemism areas**: **Brazilian sub-region**: South Brazilian dominion: Rondônia province (see Morrone 2014; fig. 12).

##### Remarks.

According to aedeagus characters, *S.ashei* sp. nov. (Fig. 1C, D) is closely related to *S.federicoescobari* (Fig. 3F), *S.martinezi* (Fig. 3I), *S.papaxibe* (Fig. 3J), *S.pygidialis* (Fig. 3O), and *S.uniplagiatus* (Fig. 3P) by having the parameres strongly asymmetric, with different shape and length (dorsal margin of left paramere with projection; ventral margin of left paramere with bilobate excavation; ventral margin of right paramere with rounded excavation), and FLP sclerite short (Fig. 1F, left), with three sets of bristles right beside it (Fig. 1E).

### Updated key to males of species of *Scybalocanthon* Martínez, 1948 (based on Silva and Valois 2019)

**Table:** 

1	Femora completely black. (*Scybalocanthonaereus* (Schmidt, 1922) (in part), *Scybalocanthonmaculatus* (Schmidt, 1920), and *Scybalocanthonkelleri* Pereira & Martínez, 1956)	**See Silva and Valois (2019): 307 for these species.**
–	Femora bicolored, central portion pale yellow, with dark spots at least on the tips (Fig. 1B)	**2**
2 (1)	Pronotum with a longitudinal dark band on midline	**See Silva and Valois (2019) for these species.**
–	Pronotum uniformly colored or, if bicolored, lacking longitudinal dark band on midline	**3**
3 (2)	Black spots on the tips of femora covering approximately 1/15 length of femora	**4**
–	Black spots on the tips of femora covering approximately 1/5 length of femora, central portion with elliptical yellow spot. (*Scybalocanthonkorasakiae* Silva, 2011, *Scybalocanthonaereus* (Schmidt, 1922) (in part), *Scybalocanthonbatesi* Vaz-de-Mello & Silva, 2017, *Scybalocanthonharoldi* Silva & Valois, 2019, *Scybalocanthonnigriceps* (Harold, 1868), *Scybalocanthondarlingtoni* (Paulian, 1939), *Scybalocanthonsexspilotus* (Guérin-Méneville, 1855), *Scybalocanthonmaculatus* (Schmidt, 1920), *Scybalocanthontrimaculatus* (Schmidt, 1922), and *Scybalocanthonmoniliatus* (Bates, 1887))	**See Silva and Valois (2019): 308, step 16, for these species.**
4 (3)	Pronotum with four black spots, two central spots with triangular shape, and one rounded spot on each side (Fig. 2G). In some individuals, one central spot can be absent (Fig. 2H). Ecuador (Fig. 5B)	***Scybalocanthonkaestneri* (Balthasar, 1939)**
–	Pronotum uniformly colored, lacking spots, or with one elliptical spot on the central portion	**5**
5 (4)	Eighth elytral stria lacking carina at the anterior portion	**See Silva and Valois (2019) for these species.**
–	Eighth elytral stria with very fine and sharp carina at the anterior portion	**6**
6 (5)	Left paramere lacking acute projection on dorsal margin and lacking bilobate excavation on ventral margin (Fig. 3D, K)	**See Silva and Valois (2019) for these species.**
–	Left paramere with acute projection on dorsal margin and bilobate excavation on ventral margin (Fig. 3F, I, J, O, P)	**7**
7 (6)	Bilobate excavation of ventral margin of left paramere wide and deep, extending at least one-half of the paramere margin in the lateral view (Figs 1D, 3I)	**8**
–	Bilobate excavation of ventral margin of left paramere short, not reaching one-half of paramere margin in the lateral view (Fig. 3F, J, P)	**See Silva and Valois (2019) for these species.**
8 (7)	Bilobate excavation of ventral margin of left paramere extending about one-half of the paramere margin in the lateral view (Fig. 3I). Ecuador and Colombia (Fig. 5C)	***Scybalocanthonmartinezi* Silva & Valois, 2019**
–	Bilobate excavation of ventral margin of left paramere extending along two-third of the paramere margin (Fig. 1D). Peru (Fig. 5C)	***Scybalocanthonashei* sp. nov.**

### New distributional data for species of *Scybalocanthon*

#### S. acrianus

Taxon classificationAnimaliaColeopteraScarabaeidae

Silva & Valois, 2019

8C88BF44-0AE1-5BFB-B0AB-86CE45E85562

##### New material examined.

In addition to those mentioned by Silva and Valois 2019.

##### Non-type material.

PERU: MADRE DE DÍOS, 15km N.E. Puerto Maldonado, Reserva Cuzco Amazonica, 12°33'S, 69°03'W, 20.VI.1989, 200m, Ashe and Leschen leg. (1♂ CMNC); Manu National Park, 15–30.VIII.1986, A. Forsyth leg. (1♂ CMNC).

##### Distribution.

Known from Brazil (Acre), Bolivia and Peru (Fig. 5B). **Endemism areas**: **Brazilian subregion**: South Brazilian dominion: Rondônia province (see Morrone 2014; fig. 12).

#### S. aereus

Taxon classificationAnimaliaColeopteraScarabaeidae

(Schmidt, 1922)

3622A32F-7944-5389-9BC3-011063683317

##### New material examined.

In addition to those mentioned by Silva and Valois 2019.

##### Non-type material.

BRAZIL: AMAZONAS, Vila Nova (1♀ CMNC)

##### Distribution.

Known from Brazil (Acre, Amazonas, Mato Grosso), Bolivia, and Peru (Fig. 5A). **Endemism areas**: **Brazilian sub-region**: South Brazilian dominion: Madeira, Ucayale, Yungas, and Rondônia provinces (see Morrone 2014; fig. 12).

#### S. kaestneri

Taxon classificationAnimaliaColeopteraScarabaeidae

(Balthasar, 1939)

3F4B8D07-AECE-5AE1-BB95-F18FF5CFEFE5

##### New material examined.

In addition to those mentioned by Silva and Valois 2019.

##### Non-type material.

ECUADOR: NAPO, P. Misahualli, 18–22.II.1983, 350m, M. Sharkey leg. (1♂ CMNC); 20km S Tena, 9–11.VII.1976, 600m, S. Peck leg. (2♀ CMNC).

##### Distribution.

Known from Ecuador (Fig. 5B). **Endemism areas**: **Brazilian sub-region**: Boreal Brazilian dominion: Napo province (see Morrone 2014; fig. 12).

#### S. pinopterus

Taxon classificationAnimaliaColeopteraScarabaeidae

(Kirsch, 1873)

80BEA269-2267-5C4C-9164-F82D8E3408D1

##### New material examined.

In addition to those mentioned by Silva and Valois 2019.

##### Non-type material.

ECUADOR: ORELLANA, Limoncocha, 10–15.III.1975, J.M. Campbell leg. (1♂ and 1♀ CMNC); Limoncocha, 13III.1976, J.M. Campbell leg. (1♂ CMNC); Limoncocha, 18–24.VI.1976, 250m, S. Peck leg. (8♂ and 8♀ CMNC); Lago Agrio, VI.1976, 250m, Martínez leg. (2♂ CMNC); PERU: HUANUCO, Tingo Maria, Universidad, XII.1974, Martínez leg. (7♂ and 16♀ CMNC); Tingo Maria, Universidad, VII.1974, Martínez leg. (2♀ CMNC); Tingo Maria, VII.1974, 700m, Bordón leg. (2♂ and 4♀ CMNC); Cucharas, Valley Huallaga, VI.1954, Felix Woytkowski leg. (1♂ CMNC); Huallaga, n.r. Tocache, 17.X.1976, 500m, J. Schunke leg. (1♂ CMNC).

##### Distribution.

Known from Ecuador and Peru (Fig. 5B). **Endemism areas**: **Brazilian sub-region**: Boreal Brazilian dominion: Napo province; South Brazilian dominion: Rondônia and Yungas provinces (see Morrone 2014; fig. 12).

## Discussion

*Scybalocanthon* now includes 24 valid species. Based on the aedeagus morphology, including endophallic sclerites, two major patterns within the genus are found: slight paramere asymmetry, namely, parameres with similar shape, but slightly different lengths; and parameres strongly asymmetric, with shapes and lengths conspicuously different. The species which have the second pattern also present the dorsal margin of left paramere with a projection (Figs 1D, 3F, I, J, O, P); ventral margin of left paramere with bilobate excavation (Figs 1D, 3F, I, J, O, P); ventral margin of right paramere with rounded excavation (Figs 1C, 3F, I, J, O, P); and FLP sclerite short, with three sets of bristles right beside it (Fig. 1E, F). Heretofore, only *S.federicoescobari*, *S.martinezi*, *S.papaxibe*, *S.pygidialis*, and *S.uniplagiatus* were known having this shape of aedeagus. We described here a new species, *S.ashei* sp. nov., which have the same characteristics mentioned above (Fig. 1C–F). However, it can be distinguished from those species by the wider bilobate excavation of the ventral margin of the left paramere, extending along two-third of the paramere margin (Fig. 1D).

Some of those species mentioned above have similar external morphology, including similarities with other species with slightly asymmetrical parameres. Characters of external morphology can also vary within the same species, such as body coloration and patterns of spots. Besides, some of these species overlap in their geographical distribution, which makes them difficult to tell apart without examining the male genitalia. According to the general external morphology and close geographical distribution, specimens of *S.ashei* sp. nov., *S.acrianus*, *S.adisi*, *S.aereus*, *S.arnaudi*, *S.chamorroi*, *S.federicoescobari*, *S.martinezi*, *S.pinopterus*, and *S.uniplagiatus* can be mistaken at first glance. All these species have distributional records in the western/central Amazon (Fig. 5A–C) and, in general, they have the pronotum and elytra uniformly colored, or with a small median spot anteriorly on pronotum (Figs 1A, 2A–F, I, K-M, O). However, *S.ashei* sp. nov. can be easily distinguished from *S.aereus*, *S.adisi*, *S.acrianus*, *S.chamorroi*, *S.arnaudi*, and *S.pinopterus* by the strongly asymmetrical parameres (Fig. 1C, D). From *S.federicoescobari*, *S.martinezi*, and *S.uniplagiatus* the genital differences have already been stated above.

**Figure 4.**
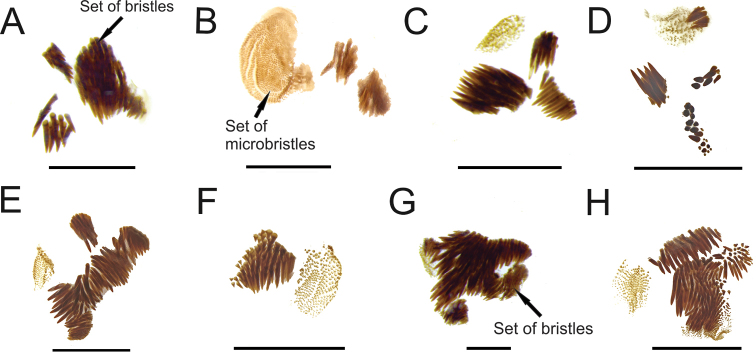
Set of bristles and microbristles of endophallus. **A***S.pygidialis* (specimen from Amazonas, Brazil) **B***S.pygidialis* (specimen from Cayenne, French Guiana) **C***S.pinopterus* (specimen from Madre de Dios, Peru) **D***S.pinopterus* (specimen from Tingo Maria, Huanuco, Peru) **E***S.pinopterus* (specimen from Tingo Maria, Huanuco, Peru) **F***S.pinopterus* (specimen from Orellana, Ecuador) **G***S.kaestneri* (specimen from Pastaza, Ecuador) **H***S.kaestneri* (specimen from Napo, Ecuador).

Silva and Valois (2019) described variations in the patterns of bristles and microbristles in endophallus of *S.pygidialis*, as follows: specimens from eastern Amazon (French Guiana and Amapá) had two sets of bristles and one set of microbristles right beside the FLP sclerite (Fig. 4B), while specimens from western Amazon (Amazonas and Roraima) had instead three sets of bristles right beside the FLP sclerite (Fig. 4A). An intraspecific variation in number of bristles and microbristles also occur at least in other species of the genus. According to Silva and Valois (2019), based on the examination of 17 males from Huánuco and Madre de Dios, Peru, *S.pinopterus* would have endophallus with three sets of bristles and one set of microbristles right beside the FLP sclerite (Fig. 4C). Four newly examined males from Tingo Maria, Huánuco, Peru, however, have four sets of bristles and a set of microbristles right beside the FLP sclerite (Fig. 4D). The parameres in these specimens (Fig. 3L) are slightly thinner and longer than those of *S.pinopterus* examined by Silva and Valois (2019) (Fig. 3K), but they have the same shape. The other five males from Tingo Maria, Huánuco, Peru, have two sets of bristles and a set of microbristles (Fig. 4E). However, there are no differences in the shape and length of parameres between these specimens (Fig. 3M) and those examined by Silva and Valois (2019) (Fig. 3K). Finally, thirteen males from Orellana, Ecuador, have only a short set of bristles and a set of microbristles (Fig. 4F), but no differences have been verified in the shape of aedeagus between these specimens (Fig. 3N) and those examined by Silva and Valois (2019) (Fig. 3K).

These observations are in agreement with the hypothesis raised by Silva and Valois (2019: 333) that the apparent allopatric distribution between populations may be artificial due to a lack of collections. Therefore, the apparent discrete differences in the sets of bristles may turn out to be an artefact and will be continuous if more specimens are examined along the geographical distribution of the species, that is, one form may intergrade into the other across this putative chain of populations, or if the forms themselves end up being indeed discrete, the frequency between them may vary among these populations.

**Figure 5.**
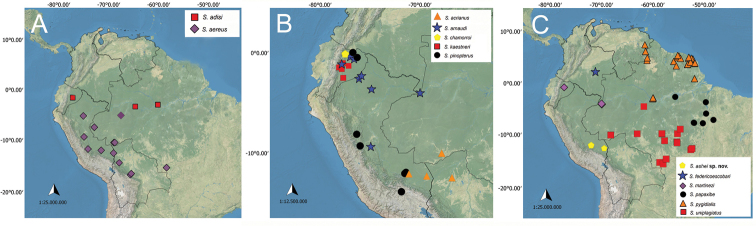
Known distributions of species of *Scybalocanthon*. **A***S.adisi* and *S.aereus***B***S.acrianus*, *S.arnaudi*, *S.chamorroi*, *S.kaestneri* and *S.pinopterus***C***S.ashei* sp. nov., *S.federicoescobari*, *S.martinezi*, *S.papaxibe*, *S.pygidialis*, and *S.uniplagiatus*.

## Supplementary Material

XML Treatment for Scybalocanthon ashei

XML Treatment for S. acrianus

XML Treatment for S. aereus

XML Treatment for S. kaestneri

XML Treatment for S. pinopterus
